# Increased Neuronal Expression of the Early Endosomal Adaptor APPL1 Replicates Alzheimer’s Disease-Related Endosomal and Synaptic Dysfunction with Cholinergic Neurodegeneration

**DOI:** 10.1523/JNEUROSCI.2331-24.2025

**Published:** 2025-06-13

**Authors:** Ying Jiang, Kuldeep Sachdeva, Chris N. Goulbourne, Martin J. Berg, James Peddy, Philip H. Stavrides, Anna Pensalfini, Monika Pawlik, Sandeep Malampati, Lauren Whyte, Balapal S. Basavarajappa, Shivakumar Subbanna, Cynthia Bleiwas, John F. Smiley, Paul M. Mathews, Ralph A. Nixon

**Affiliations:** ^1^Center for Dementia Research, Nathan S. Kline Institute for Psychiatric Research, Orangeburg, New York 10962; ^2^Department of Psychiatry, New York University Grossman School of Medicine, New York, New York 10016; ^3^Emotional Brain Institute, Nathan S. Kline Institute for Psychiatric Research, Orangeburg, New York 10962, and; ^4^Departments of Cell Biology, New York University Grossman School of Medicine, New York, New York 10016; ^5^Neuroscience Institute, New York University Grossman School of Medicine, New York, New York 10016

**Keywords:** AD, APP-βCTF, APPL1, ChAT^+^ neuron, endosome, rab5

## Abstract

Endosomal system dysfunction within neurons is a prominent early feature of Alzheimer's disease (AD) pathology. Multiple AD risk factors are regulators of endocytosis and known to cause hyperactivity of the early endosome small GTPase rab5, resulting in neuronal endosomal pathway disruption and cholinergic neurodegeneration. Adaptor protein containing Pleckstrin homology domain, Phosphotyrosine binding domain, Leucine zipper motif (APPL1), an important rab5 effector protein and signaling molecule has been shown in vitro to interface between endosomal and neuronal dysfunction through a rab5-activating interaction with the BACE1-generated C-terminal fragment of amyloid precursor protein (APP-βCTF), a pathogenic APP fragment generated within endosomal compartments. To understand the contribution of APPL1 to AD-related endosomal dysfunction in vivo, we generated a transgenic mouse model overexpressing human APPL1 within neurons (Thy1-APPL1). Strongly supporting the important endosomal regulatory roles of APPL1 and their relevance to AD etiology, Thy1-APPL1 mice (both sexes) develop enlarged neuronal early endosomes and increased synaptic endocytosis due to increased rab5 activation. We demonstrated pathophysiological consequences of APPL1 overexpression, including functional changes in hippocampal long-term potentiation (LTP) and long-term depression (LTD), degeneration of large projection cholinergic neurons of the basal forebrain, and impaired hippocampal-dependent memory. Our evidence shows that neuronal APPL1 elevation modeling its functional increase in the AD brain induces a cascade of AD-related pathological effects within neurons, including early endosome anomalies, synaptic dysfunction, and selective neurodegeneration. Our in vivo model highlights the contributions of APPL1 to the pathobiology and neuronal consequences of early endosomal pathway disruption and its potential value as a therapeutic target.

## Significance Statement

Neuronal endosome dysfunction appears early in Alzheimer's disease (AD) and is linked to memory loss. Genes and risk factors associated with AD often increase rab5 activity, a protein that disrupts endosomal signaling when hyperactivated. APPL1, a key rab5 partner, worsens this dysfunction via its interaction with APP-βCTF, a protein fragment associated with AD. To explore APPL1's role, we created a genetically modified mouse that overexpresses APPL1 in neurons. This model provides the first in vivo evidence that APPL1 overexpression triggers key AD-like effects: rab5 hyperactivation, enlarged early endosomes, loss of cholinergic neurons, reduced synaptic plasticity in memory-related brain regions, and memory deficits. These findings highlight APPL1's role in AD pathogenesis and its potential as a therapeutic target.

## Introduction

Endosomal pathway dysfunction is the earliest known cytopathology of Alzheimer's disease (AD), with endosomal anomalies emerging years, possibly decades, before β-amyloid and tau tangles (Cataldo et al., 2000; Colacurcio et al., 2018; Pensalfini et al., 2020; Fahimi et al., 2021). The apolipoprotein E (apoE) e4 allele, the major genetic risk factor for late onset AD, accelerates development of neuronal endosomal alterations (Cataldo et al., 2000). Similar changes were seen in apoE e4 mouse models without β-amyloid or tau pathology (Nuriel et al., 2017; Peng et al., 2024). Trisomy 21 Down syndrome (DS), which leads to AD in adulthood, is associated with early endosome disruption and early emerging cholinergic neurodegeneration in the basal forebrain due to intracellular accumulation of APP-βCTF, a C-terminal fragment of amyloid precursor protein (APP; Jiang et al., 2016, 2019; Kim et al., 2016; Colacurcio et al., 2018; Lauritzen et al., 2019). APP-βCTF levels are elevated in human AD brain (Pera et al., 2013; Kim et al., 2016) and in AD models where they contribute directly to early endosome dysfunction, disrupted endosomal trafficking, and neuronal loss (Jiang et al., 2010, 2016, 2019; Lauritzen et al., 2016; Bourgeois et al., 2018; Colacurcio et al., 2018).

Both APP-βCTF and rab5 proteins on endosome membrane bind to APPL1 (Adaptor Protein containing Pleckstrin homology domain, Phosphotyrosine binding domain, and Leucine zipper motif) via the phosphotyrosine binding domain and pleckstrin homology domain, respectively, interactions that lead APPL1 to accumulate at rab5-containing endosomal membranes (Miaczynska et al., 2004; Mao et al., 2006; Zhu et al., 2007; Kim et al., 2016; Diggins and Webb, 2017; Xu et al., 2018; Hiragi et al., 2022). APPL1 stabilizes the GTP-bound, endocytic-active form of rab5, leading to accelerated endocytosis, early endosome enlargement, and impaired transport of endosomes in primary cortical neurons (Kim et al., 2016). Given that directly hyperactivating rab5 in vivo in mice via modest neuronal overexpression of rab5 recapitulates AD-related endosome anomalies and their neurodegenerative consequences (Kim et al., 2016; Pensalfini et al., 2020), we investigated whether APPL1 stabilization of active rab5 induces similar pathologies, thereby linking APPL1 elevation seen in AD (Johnson et al., 2022) and early endosome pathobiology.

APPL1 interacting with rab5 at the early endosomal membrane modulates endocytic trafficking while interfacing with various structural and signaling proteins (Miaczynska et al., 2004; Mao et al., 2006; Zhu et al., 2007; Diggins and Webb, 2017; Hiragi et al., 2022). Notably, APPL1 modulates Akt signaling (Majumdar et al., 2011; Bohdanowicz et al., 2012; Wang et al., 2016; Goto-Silva et al., 2019) and mediates nerve growth factor (NGF) signaling through TrkA (Lin et al., 2006) to support cholinergic neurons. Following rab5-GTP hydrolysis, APPL1 dissociates from early endosomal membranes and translocate to the nucleus to regulate gene expression (Rashid et al., 2009; Zoncu et al., 2009). APPL1 also modulates hippocampal synaptic plasticity (Formolo et al., 2022) including controlling *N*-methyl-*d*-aspartate receptor (NMDAR)-dependent potentiation that facilitates extinction memory retrieval (Hua et al., 2023).

To examine in vivo the interface between APPL1 function and the diverse endosomal and neurodegenerative consequences attributed to early endosome pathobiology in AD, we generated a novel transgenic mouse model that overexpresses APPL1 in neurons (Thy1-APPL1 mice). With modest APPL1 overexpression (two-fold), this Thy1-APPL1 model replicates the AD-related endosome phenotype mediated by rab5 hyperactivation and its downstream pathological consequences on neuronal function, including those at hippocampal synapses. Importantly, the number of choline acetyltransferase (ChAT)-positive neurons in the medial septal nucleus (MSN) was reduced in Thy1-APPL1 mice. Hippocampal-dependent memory, measured by the novel object recognition (NOR) test, was impaired in Thy1-APPL1 mice. These in vivo findings support the idea that APPL1 recruitment to early endosomes is sufficient to replicate endosomal, functional, and neurodegenerative changes induced by elevated APP-βCTF levels and rab5 overactivation in AD and related animal models (Jiang et al., 2016, 2019, 2022; Pensalfini et al., 2020; Peng et al., 2024).

## Materials and Methods

### Thy1-APPL1 transgene construct, generation of transgenic mice, and genotyping

Transgenic mice overexpressing human APPL1 (NM_012096) were generated by Ingenious Targeting Laboratory using the pTSC21 vector consisting of a murine Thy1 expression cassette (Pensalfini et al., 2020) and a Flag-tag for identification. Two founder mice (M1 and F2) were obtained on a mixed background of 129SvEC and C57BL/6, which were then crossed with C57BL/6 mice (JAX stock number 000664, The Jackson Laboratory) for over 10 generations to establish the line used in this study; offsprings from founder F2 mice were used for the current study. Thy1-APPL1 mice genotyping was accomplished by PCR using the following primer pair: forward-5′ ATGAAGTCACCCAGCAGGGAG and reverse 5′ AGGTCAGGTGTGTTGCTGCAC. Each reaction consisted of H_2_O (23 µl), 2× GO Taq master mix (M7122, Promega), primer pair (0.5 µl each), and tail DNA (2 µl of a 0.05–0.1 mg/ml solution). The PCR conditions were as follows: 94°C for 3 min, followed by 30 cycles of 94°C for 30 s, 58°C for 30 s, and 72°C for 30 s, with a final extension step at 72°C for 5 min. The reaction mixes were then loaded onto agarose gels; Thy1-APPL1 mice were positively identified by a band presented at or above 500 bp. Thy1-APPL1 mice bred normally with the expected Mendelian ratio of offsprings, with normal body weight and lifespan. Throughout, nontransgenic (non-Tg) wild-type littermate mice were used as controls.

Mouse experimentation and animal care were approved by the Institutional Animal Care and Use Committee (IACUC) of the Nathan S. Kline Institute. The approval is renewed every 3 years, and the current protocol number is AP2024-746. Mice were housed in the NKI Animal facility and kept under a 12 h day and night cycle, at temperatures of ∼70°F (±2°F) and humidity between 40 and 60%. Both male and female mice were used.

### Immunocytochemistry and Western blot analysis

Thy1-APPL1 mice and age-matched non-Tg were anesthetized with a combination of ketamine (100 mg/kg BW) and xylazine (10 mg/kg BW) and transcardially perfused with 0.9% NaCl in 0.1 M phosphate-buffered, pH 7.4, as described previously (Yang et al., 2014). One hemibrain was frozen and stored at −80°C for biochemical analysis (see below), while the other hemibrain was drop-fixed in 4% paraformaldehyde (PFA) in 0.1 M sodium cacodylate buffer, pH 7.4 (Electron Microscopy Sciences) for immunolabeling as previously described (Pensalfini et al., 2020; Jiang et al., 2022). After >48 h of fixation, 40-μm-thick vibratome brain tissue sections from various brain regions were collected and washed three times with antibody dilution buffer containing bovine serum albumin (BSA; 1%; Sigma), saponin (0.05%; Sigma), and normal horse serum (NHS; 1%; Thermo Fisher) in Tris-buffered saline (TBS), pH 7.4 and then blocked with 20% NHS in TBS for 1 h at room temperature before incubation with commercial antibodies against rab5 (Abcam, catalog #ab18211, RRID:AB_470264; 1:500; Kim et al., 2016; Pensalfini et al., 2020), rab5-GTP (active rab5: NewEast Biosciences, catalog #26911, RRID:AB_2617182; 1:50; Kim et al., 2016; Pensalfini et al., 2020), ChAT (Millipore, catalog #AB144P, RRID:AB_2079751; 1:250; Jiang et al., 2016; Pensalfini et al., 2020), APPL1 (Proteintech, catalog #12639-1-AP, RRID:AB_2289669; 1:500; Kim et al., 2016), and the Flag-tag (Thermo Fisher Scientific, catalog #MA1-91878-D488, RRID:AB_2537621; 1:500), NeuN (Millipore, catalog #MAB377, RRID:AB_2298772; 1:2000; Lee et al., 2018), either alone or in combination and visualized with either biotinylated secondary antibodies (Vector Laboratories; catalog #BA-9200, BA-1000 and BA-6000; 1:500) or fluorescence-conjugated secondary antibodies (Thermo Fisher; catalog #A10037, A21206 and A21099; 1:500), as described in our previous studies (Pensalfini et al., 2020; Jiang et al., 2022). An additional mouse monoclonal antibody against APPL1 (Novus, catalog #NBP2-46536; RRID:AB_3083472; 1:500) was used for coimmunolabeling with a rabbit polyclonal rab5 antibody (Abcam, catalog #ab218624). Confocal images were collected using a Zeiss LSM880 laser confocal microscope and Zen 2.1-Sp3 software. Rab5 and rab5-GTP positive puncta, including intensity, number, average size, and total area per cell, were determined by Fiji/ImageJ 2.3.0 (https://imagej.net/Fiji). Three or more mice per genotype were analyzed, and 20–30 neurons from each mouse were quantified as previously reported (Pensalfini et al., 2020; Jiang et al., 2022). For protein analyses, mouse hemibrains were homogenized in 1:10 (brain weight: buffer), pH 7.4, buffer containing 250 mM sucrose, 20 mM Tris-HCl, 1 mM EDTA, 1 mM EGTA, protease/phosphatase inhibitors (Pensalfini et al., 2020; Jiang et al., 2022), and Western blot analyses as described previously and were performed with antibodies against APP (C1/6.1; 1 µg/ml; Jiang et al., 2010), APP-βCTF (M3.2; 2 µg/ml; Morales-Corraliza et al., 2009), APPL1 (Proteintech; 1:1,000), rab5 (Abcam; 1:2,000), synaptophysin (Sigma-Aldrich, catalog #S5768, RRID:AB_477523; 1:2,000; Pensalfini et al., 2020), phosphorylated tau [paired-helical filament tau, phosphorylated at Ser396 and Ser404 site (PHF1); 1:1,000, kind gift from P. Davies; Pensalfini et al., 2020], and total tau protein (Agilent, catalog #A0024, RRID:AB_10013724; 1:5,000; Pensalfini et al., 2020). All the secondary antibodies for Western blot analyses were used according to the manufacturer's recommendations (Jackson ImmunoResearch Laboratories). As an internal loading control, total protein on each blot was determined by Revert 700 Total Protein Stain (Licor; catalog #926-11021; Wijewantha et al., 2024). Digital gel imager (Syn-gene G:Box XX9) was used to capture both the total protein stain and the ECL images from each blot. Band intensities on each blot were quantified with a combination of Fiji/ImageJ 2.3.0 and Multi Gauge (Fujifilm; V3.0) software.

### ELISA of Aβ40 and Aβ42 of DEA extracts from brain tissue of Thy1-APPL1 and non-Tg mice

Aβ40 and Aβ42 levels were determined by sandwich ELISA according to previously published protocols (Schmidt et al., 2012; Jiang et al., 2016). Briefly, brain homogenates were extracted with 0.4% diethylamine (Sigma, catalog #D3131)/100 mM NaCl solution (1:1 of v/v) and then processed further with a tissue grinder before centrifugation at 43,000 rpm for 1 h at 4°C in a Beckman Coulter TLA 100.3 rotor. The collected supernatants were mixed with 10% volume of 0.5 M Tris buffer, pH 6.8, and 100 µl of the buffered samples was then loaded into ELISA plates coated with specific anti-Aβ40 or Aβ42 antibodies as reported previously (Schmidt et al., 2005; Jiang et al., 2016). Horseradish peroxidase (HRP) conjugated secondary antibody (M3.2, 1:1,000; Choi et al., 2009; Jiang et al., 2016) was used for both Aβ40 and Aβ42 detections, and a multimode plate reader (SpectraMax M5, Molecular Devices) was used to read the optical density (O.D.) at 450 nm. The level of Aβ40 or Aβ42 was presented in fmol/g of brain tissue, calculated according to a standard curve using synthetic mouse Aβ40 or Aβ42 peptides (American Peptide; Choi et al., 2009; Jiang et al., 2016; Pensalfini et al., 2020).

### Stereological counting of medial septal nucleus ChAT-immunoreactive neurons

For ChAT immunolabeling, every third consecutive vibratome brain tissue section containing MSN was treated with 3% H_2_O_2_ before blocking with 20% NHS in TBS for 1 h at room temperature, followed by incubation with anti-ChAT antibody (MilliporeSigma; 1:250) as described previously (Jiang et al., 2016, 2022). Visualization was achieved using diaminobenzidine (DAB; catalog #SK-4100; Vector Laboratories) after incubation with a biotinylated secondary antibody (1:500; Vector Laboratories) and the Vectastain ABC kit (Vector Laboratories, catalog #PK6105). To quantify ChAT-positive neurons in the MSN, the optical fractionator method was used combined with ImageJ software as previously described (West et al., 1991; Smiley et al., 2012; Jiang et al., 2016, 2022). Cell counts were corrected for the *z*-axis section thickness, which was measured three times per section. The dissector size and density were optimized to maintain a coefficient of error below 0.1.

### Electrophysiology

Thy1-APPL1 and non-Tg littermate mice (*n* = 5 for each genotype) were used to measure long-term potentiation (LTP) and long-term depression (LTD) in the hippocampal CA1 as detailed in previous studies (Subbanna et al., 2013; Pensalfini et al., 2020). Mice were killed by cervical dislocation and the hippocampi were immediately collected, followed by sectioning to 400 µm slices. The hippocampal slices were then placed into a recoding chamber filled with artificial cerebrospinal fluid (in mM: 124 NaCl, 4.4 KCl, 1.0 Na_2_HPO_4_, 25 NaHCO_3_, 2 CaCl_2_, 2 MgSO_4_, 10 glucose, osmolarity between 290 and 300 Osm/L) at 29°C with 95% O_2_ and 5% CO_2_. CA1 field excitatory postsynaptic potentials (fEPSPs) were recorded by placing the stimulating and recording electrodes in the hippocampal CA1 stratum radiatum. LTP was induced by theta-burst stimulation (TBS, 4 pulses at 100 Hz, with the bursts repeated at 5 Hz, and each tetanus including three 10-burst trains separated by 15 s), and LTP responses were recorded for 2 h. LTD was induced by low-frequency stimulation (LFS, 1 Hz for 900 s) and LTD responses were recorded for 80 min. For both LTP and LTD experiments, a 10 min baseline was recorded at 1 min intervals with stimulus to evoke a response ∼35% of the maximum evoked response. Both LTP and LTD fEPSP slopes were expressed as percentage of baseline (the average value 10 min before stimulation). The results are expressed as both fEPSP slopes and combined bar graphs of the averages of fEPSP slopes at indicated time points (mean ± SEM) with individual mouse data as previously reported (Subbanna et al., 2013; Pensalfini et al., 2020).

### Electron microscopy and post-embedding immunogold EM

Electron microscopy (EM) and post-embedding immunogold EM were performed on brain sections as previously described (Yang et al., 2009; Pensalfini et al., 2020). Mice were transcardially perfused with a solution of 4% PFA and 2% glutaraldehyde in 0.1 M sodium cacodylate buffer, pH 7.4. Fixed tissue was cut into 80-μm-thick sagittal vibratome sections and postfixed in 1% osmium tetroxide. Following alcohol dehydration, tissue sections were infiltrated with increasing concentrations of Spurr resin and then embedded flat in Aclar sheets. Regions of interest for ultrastructural analyses, including the prefrontal cortex and hippocampal CA1, were excised, and 50 nm ultrathin sections were prepared and stained with uranyl acetate and lead citrate. The thin sections were then viewed using a Thermo Fisher Talos L120C transmission electron microscope operating at 120 kV. For endosome quantification, ∼60 EM images (17,500×) per mouse were acquired. The images contained dendritic and synaptic profiles in the proximity (within 5–10 µm) of the neuronal soma located within the pyramidal cell layer V of the prefrontal cortex (*n* = 7 for both non-Tg and Thy1-APPL1 mice) and CA1 of the hippocampus (*n* = 4 for both genotypes). The average size and circumference of endosomes for each mouse genotype were determined using Fiji/ImageJ (Schindelin et al., 2012). For immunogold EM, ultrathin tissue sections were mounted on nickel grids, air-dried, and then etched for 5 min with 1% sodium metaperiodate in PBS. After washing with filtered double-distilled water, the ultrathin tissue sections were incubated with 1% BSA in PBS for 2 h, followed by overnight incubation with the combination of mouse anti-APPL1 antibody (dilution of 1:2) and rabbit anti-Rab5 antibody (dilution of 1:2) in a humidified chamber at 4°C. These sections were incubated with gold-conjugated secondary anti-mouse (size 10 nm) and anti-rabbit (size 6 nm) antibodies for 2 h at room temperature before imaging. For colocalization quantification, ∼40 EM images (22,000× and 57,000×) per mouse were acquired from the pyramidal cell layer V of the prefrontal cortex and CA1 of the hippocampus of non-Tg and Thy1-APPL1 mice (*n* = 3 each genotype). Rab5-immunoreactive endosomes, as well as rab5/APPL1 dual-immunoreactive endosomes, were counted manually, with results presented as percentage of rab5^+^/APPL1^+^ endosomes versus total rab5^+^ endosomes.

### Synaptosome isolation

Synaptosomes were isolated from non-Tg and Thy1-APPL1 mouse hippocampi (*n* = 6 mice per genotype). For hippocampal synaptosomes, hippocampi were homogenized in 250 µl of homogenization buffer (0.32 mmol/L sucrose, 0.1 mmol/L CaCl_2_, 1 mmol/L MgCl_2_) with protease and phosphatase inhibitors (5 µg/ml Pepstatin A, 5 µμ Leupeptin, 1 mM AEBSF, 1 µg/ml microcystin) as previously described (Louneva et al., 2008; Pensalfini et al., 2020). Samples were adjusted to contain 1.25 M sucrose and sequentially overlaid with 1.0 M sucrose solution containing 0.1 mM CaCl_2_, followed by a layer of homogenization buffer. Tubes were centrifugated at 100,000 × *g* for 3 h at 4°C using a SW55Ti rotor (Beckman Coulter). Synaptosomes enriched in the interface between 1.0 and 1.25 M sucrose were collected and split equally between synaptic vesicle endocytosis (SVE) analyses (see below) and biochemical analyses. For biochemistry, the synaptosomes were washed twice with ice-cold 0.1 mM CaCl_2_, followed by centrifugation at 50,000 × *g* for 30 min at 4°C in a SW55Ti rotor. Washed pellets were dissolved in 8 M urea prior to further biochemical analyses.

### Synaptic vesicle endocytosis

Synaptosomes from non-Tg and Thy1-APPL1 mouse hippocampi (*n* = 4–6 mouse each genotype) were used for SVE analysis (Daniel et al., 2012; Sellgren et al., 2019; Shekarabi et al., 2019). Synaptosomes from the 1.0–1.25 M sucrose interface were resuspended in sucrose/EDTA/Tris buffer (SET buffer: 0.32 M sucrose, 1 mM EDTA, 5 mM Tris, pH 7.4) and centrifugated at 20,000 × *g* for 10 min at 4°C in a SW55Ti rotor, followed by resuspension in SET buffer. The resuspended synaptosomes were aliquoted and slowly frozen with 5% DMSO in SET buffer to preserve integrity as described (Daniel et al., 2012). On the day of the assay, aliquots of synaptosomes were thawed in a 37°C water bath for 80 s and mixed with 1 ml of ice-cold SET buffer, followed by centrifugation at 18,900 × *g* for 2 min at 4°C to pellet the synaptosomes. The synaptosome pellets were again suspended in 300 µl of SET buffer with a wide-orifice 1 ml pipette tip. After protein quantification, 2 µg of synaptosomes were suspended in SET buffer containing 250 µM dithiothreitol to prevent clumping and loaded per well into a polyethyleneimine-coated 96-well glass-bottom microplate. The 96-well plate with synaptosomes was then centrifugated in a microplate centrifuge (Sigma, 4-15C) at 1,500 × *g* for 30 min at 4°C to seed the synaptosomes onto the plate before incubation in HBK buffer (HEPES-buffered Krebs-like buffer; in mM: 143 NaCl, 4.7 KCl, 1.3 MgSO_4_, 1.2 CaCl_2_, 20 HEPES, 0.1 NaH_2_PO_4_, and 10 d-glucose, pH 7.4) at 37°C for 15 min. The synaptosomes were then labeled with 5 µM of CellTracker Green CMFDA (5-chloromethyl fluorescein diacetate; Thermo Fisher; catalog #C7025) to label viable synaptosomes for 30 min at 37°C, followed by an 8 min pulse with 4 µM of the endocytic dye FM4-64 [*N*-(3-triethylammoniumpropyl)-4-(6-(4-(diethylamino) phenyl) hexatrienyl) pyridinium dibromide, Thermo Fisher; T13320]. Labeled synaptosomes were then washed with 1 mM of Advasep-7 (Biotium, 70029) for 2 min to remove extracellular dye, before fixation with 2% PFA in HBK buffer for 30 min on ice, followed by imaging at 40× magnification (with 3× zoom) using a LSM880 confocal microscope. For quantification, 25–30 synaptosome images were taken from each mouse, and the intensity of FM4-64 and CMFDA within synaptosomes was measured with CellProfiler (https://cellprofiler.org) to obtain the ratio of FM4-64/CMFDA per synaptosome in non-Tg and Thy1-APPL1 mice (*n* = 4–6). Additional immunofluorescence staining with anti-rab5 antibody (Abcam, 1:200) was performed after CMFDA intake while avoiding dislodging the attached synaptosomes. The intensity of the rab5 and CMFDA signals was measured with ImageJ to determine the ratio of rab5/CMFDA in non-Tg and Thy1-APPL1 mice (*n* = 3).

### GTP-agarose beads pull-down assay

Synaptosomes isolated as described above from non-Tg and Thy1-APPL1 mouse hippocampus (*n* = 3 mice per genotype) were used for a GTP-agarose beads pull-down assay (Zhang et al., 2013; Fang et al., 2017; Pensalfini et al., 2020). Briefly, synaptosome pellets were resuspended in GTP-agarose lysis/wash buffer (50 mM Tris-HCl, pH7.5; 250 mM NaCl, 5 mM Mg acetate, 0.5% Triton X-100, and protease inhibitors), incubated on ice for 30 min, and then sonicated prior to a BCA protein assay. Then, 200 µg of the synaptosome lysates was added into 300 µl of prewashed GTP-agarose beads (Sigma, catalog #G9768). The synaptosome and bead mixtures were incubated overnight at 4°C with rotation and then washed three times with wash buffer before being boiled for 10 min in 2× Laemmli sample buffer. As reference, 10 µg of synaptosome lysate was used as “Input” for Western blot analysis as described above.

### Novel object recognition

NOR was performed according to our previous publications (Pensalfini et al., 2020; Jiang et al., 2022). A multiple unit open field with four activity chambers and a digital video camera were used to record the activity of the mice inside each chamber. Then, 12–13-month-old non-Tg and Thy1-APPL1 mice (*n* = 10 for both genotypes) were used for the test. Mice were habituated by freely exploring the chamber for 5 min per day for 2 d. On Day 3, a training session was conducted by placing an individual mouse together with two identical objects (familiar) for 10 min, followed by one familiar and one novel object for 10 min after a 3 h interval. The recorded videos were analyzed with CowLog 3 (Hänninen and Pastell, 2009) to determine the exploration time of each mouse. The results were presented as recognition index (RI), defined as time exploring the novel object versus the sum of the time exploring both the novel and familiar objects, as previous described (Pensalfini et al., 2020; Jiang et al., 2022).

### Experimental design and statistical analysis

All quantitative data were subjected to a two-tailed unpaired Student's *t* test for a single comparison and a one-way ANOVA analysis for multiple comparisons, with post hoc Tukey's analysis, using GraphPad Prism 8.0.1. Data is represented in bar graphs as the Mean ± SEM, with individual data points for each mouse in the study group. Statistical significance is represented by asterisks: *p < 0.05, **p < 0.01, ***p < 0.001. Sample sizes were determined based on similar experimental procedures in our previous studies (Pensalfini et al., 2020; Jiang et al., 2022). Initial age studies showed that the basal forebrain cholinergic neurons (BFCN) loss in the Thy1-APPL1 mice was first apparent at 12–13 months of age; therefore, unless specifically stated in the text, most presented findings are of non-Tg and Thy1-APPL1 mice at 12–13 months of age.

## Results

### APPL1 overexpression in the brains of transgenic mice

**Figure 1.**
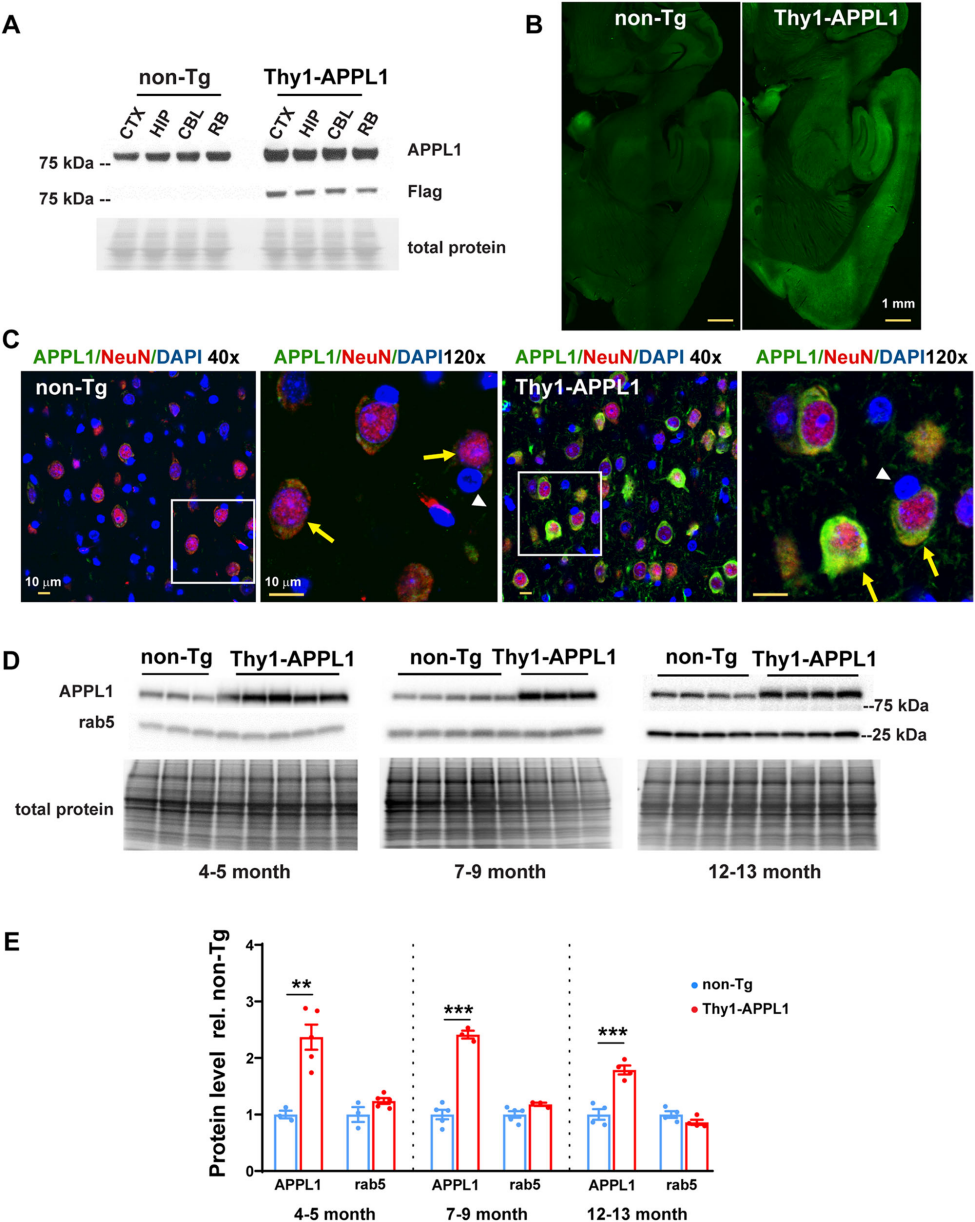
APPL1 transgene expression in the brain. ***A***, Representative Western blot comparing APPL1 expression in various brain regions (CTX, cortex; HIP, hippocampus; CBL, cerebellum; RB, remaining brain) from 4- to 5-month-old nontransgenic littermate (non-Tg) and Thy1-APPL1 transgenic (Thy1-APPL1) mice. Overexpressed flag-tagged APPL1 proteins were detected in all brain regions in Thy1-APPL1 mice. ***B***, Representative brain section immunolabeling with an anti-APPL1 antibody at low magnification (scale bar, 1 mm) demonstrates the APPL1 overexpression patterns in various brain regions of Thy1-APPL1 mice. ***C***, Representative immunofluorescent images at low (40×) and high (120×) magnification show APPL1 (green) and NeuN (red) with DAPI (blue) staining in cortex layer V (scale bar, 10 µm; yellow arrows indicate neurons, white arrowheads identify non-neuronal cells; genotype as indicated). ***D***, Western blots probed with anti-APPL1 and anti-rab5 antibodies of both non-Tg and Thy1-APPL1 brain homogenate prepared from mice of the indicated ages. ***E***, Western blot analysis quantification showing increased APPL1 in the brain homogenates of Thy1-APPL1 mice compared with non-Tg mice at the indicated ages. **p < 0.01, ***p < 0.001, two-tailed, unpaired *t* test. Data shown as mean ± SEM.

APPL1 transgenic lines under the Thy1 promoter were generated, and an experimental line was chosen and backcrossed prior to expansion and experimentation (Thy1-APPL1). APPL1 overexpression was confirmed by Western blot analysis (Fig. 1*A*). Greater APPL1 immunolabeling was seen broadly in multiple brain regions (including cortex, hippocampus, cerebellum) when comparing Thy1-APPL1 with non-Tg mice (Fig. 1*A*,*B*), with a neuronal expression shown by coincident immunolabeling of APPL1 (green) and NeuN (red; Fig. 1*C*; cortex). Increased immunolabeling for APPL1 is evident in neurons when comparing Thy1-APPL1 and non-Tg brain tissue (Fig. 1*C*, comparing yellow APPL1 labeling in NeuN-positive neurons, yellow arrows). Non-neuronal cells identified by DAPI nucleus staining showed similar levels of APPL1 immunolabeling in both Thy1-APPL1 and non-Tg brain tissue (Fig. 1*C*, white arrow heads). Western blot analysis of brain cortex homogenate across three age cohorts ranging from 4–5 to 12–13 months of age showed an approximate doubling of brain APPL1 levels in the Thy1-APPL1 mice compared with non-Tg littermates (Fig. 1*D*,*E*; *n* = 3–5 for non-Tg and Thy1-APPL1; *t*_(6)_ = 4.550, *p* = 0.00392 for 4–5-month-old mice; *t*_(6)_ = 11.5, *p* = 0.000025 for 7–9-month-old mice; *t*_(6)_ = 6.387, *p* = 0.000693 for 12–13-month-old mice; two-tailed, unpaired *t* test). Detected with a pan-rab5 antibody that does not differentiate active from inactive forms of the protein, rab5 protein levels in brain homogenates were unchanged when comparing APP1 overexpressing mice to non-Tg (Fig. 1*D*,*E*). Thus, Thy1-APPL1 mice show neuronal specific APPL1 overexpression across many brain regions, an increase that does not result in a change in overall brain rab5 protein levels.

### Thy1-APPL1 transgenic mice have early endosomal abnormalities

**Figure 2.**
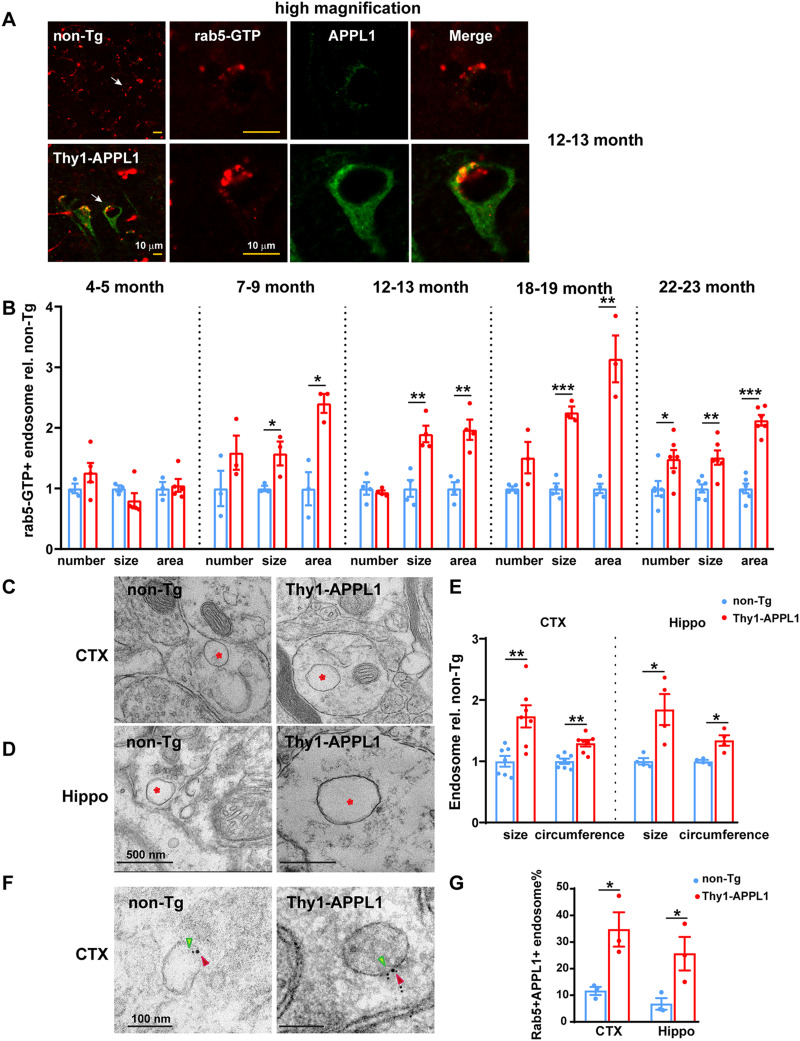
Early endosome alterations in Thy1-APPL1 mice. ***A***, Representative immunofluorescent images of active, GTP-bound rab5 (rab5-GTP; red) and APPL1 (green) in neurons (layer V, prefrontal cortex) of non-Tg and Thy1-APPL1 mice at 12–13 months of age. Shown are images at lower magnification (40×, merged). The white arrow indicating the neuron shown at higher magnification for individual and merged immunofluorescent signal. Scale bar, 10 µm. ***B***, Quantification of the average number, size, and total area of Rab5-GTP immunolabeled endosomes per cortical neuron in non-Tg versus Thy1-APPL1 mice at the indicated ages. Representative electron microscopy images containing dendritic profiles in layer V of prefrontal cortex (***C***) and hippocampus (***D***) regions and (***E***) quantification of average size and circumference of endosomes in both cortex and hippocampi showing the enlargement in Thy1-APPL1 compared with non-Tg mice aged at 12–13 months (red asterisk indicating endosome; 7 mice each genotype for cortex and 4 mice each genotype for hippocampi were used for quantification. Scale bar, 500 nm). Sixty images/mouse were quantified in ***E***; for cortex sections, a total of 2,635 endosomes from 14 mice were counted, averaging 188 endosomes per mouse; for hippocampal sections, a total of 1,594 endosomes were counted from 8 mice, with an average of 199 endosomes per mouse. ***F***, Representative immunoelectron microscopy images containing dendritic profiles in layer V of prefrontal cortex labeled with rabbit anti-rab5 and mouse anti-APPL1 followed by gold-conjugated secondary anti-rabbit (6 nm, green arrow) and anti-mouse (10 nm, red arrow) in non-Tg and Thy1-APPL1 mice. Scale bar, 100 nm. ***G***, Percentage of rab5/APPL1 double-positive endosomes versus total rab5-positive endosomes in the dendritic region of the cortex and hippocampus significantly increased in Thy1-APPL1 mice (3 mice per genotype. 35–40 images/mouse were used for counting; for cortex sections, a total of 130 endosomes were counted from 6 mice, with an average of 22 endosomes per mouse; for hippocampal sections, a total of 121 endosomes were counted from 6 mice, with an average of 20 endosomes per mouse). *p < 0.05, **p < 0.01, ***p < 0.001, two-tailed, unpaired *t* test. Data is shown as mean ± SEM.

Our previous study showed that APPL1 is a molecular intermediary between APP-βCTF-driven endosomal alterations and rab5, required for increased APP-βCTF levels to drive such early endosome dysfunctions as enlargement and disrupted trafficking in a cell model (Kim et al., 2016). One goal of the current study using the Thy1-APPL1 mice was to determine whether an increase in APPL1 is sufficient to drive the same range of early endosome pathologies in vivo, directly testing whether APPL1 can play a primary role in AD-relevant early endosomal alterations. Immunolabeling with an anti-rab5 antibody that only detects the active, early endosome membrane-associated, GTP-bound state of rab5 (rab5-GTP; Mani et al., 2016; Pensalfini et al., 2020) was done comparing cortical pyramidal neurons between Thy1-APPL1 and non-Tg mice (Fig. 2*A*). A significantly higher level of active rab5 immunolabeling was seen in cortical layer V neurons of Thy1-APPL1 mice compared with non-Tg littermates. Representative immunolabeling at low magnification of 12-month-old non-Tg and Thy1-APPL1 mice shows the coincidence of rab5-GTP (red) and APPL1 (green; Fig. 2*A*, left panels). Higher magnification of a single neuron illustrates typical rab5-GTP and APPL1 immunolabeling in both genotypes (Fig. 2*A*). There was also significantly higher colocalization of APPL1 with rab5-GTP in Thy1-APPL1at 12–13 month of age, with Pearson's *R* = 0.161 in non-Tg versus *R* = 0.303 in Thy1-APPL1 mice (*n* = 4 each for non-Tg and Thy1-APPL1 mice, *t*_(6)_ = 3.986, *p* = 0.0072, two-tailed, unpaired *t* test), consistent with overexpressed APPL1 leading to the recruitment of rab5 to early endosomes.

The quantitative data across the life span of non-Tg and Thy1-APPL1 mice are summarized in Figure 2*B* (*n* = 3–6 each age and genotype; *t*_(6)_ = 1.198, *p* = 0.276; *t*_(6)_ = 1.201, *p* = 0.273; *t*_(6)_ = 0.318, *p* = 0.761; for number, size, and area of 4–5-month-old mice; *t*_(4)_ = 1.457, *p* = 0.219; *t*_(4)_ = 2.844, *p* = 0.0467; *t*_(4)_ = 4.467, *p* = 0.0111 for number, size, and area of 7–9-month-old mice, respectively; *t*_(6)_ = 0.577, *p* = 0.585; *t*_(6)_ = 4.614, *p* = 0.00364; *t*_(4)_ = 4.930, *p* = 0.00263 for number, size, and area of 12–13-month-old mice; *t*_(5)_ = 2.307, *p* = 0.0691; *t*_(5)_ = 9.717, *p* = 0.000196; *t*_(5)_ = 6.356, *p* = 0.00142; for number, size, and area of 18–19-month-old mice; *t*_(10)_ = 2.515, *p* = 0.0306; *t*_(10)_ = 3.737, *p* = 0.00386; *t*_(10)_ = 9.818, *p* = 0.000002; for number, size, and area of 22–23-month-old mice; two-tailed, unpaired *t* test). Interestingly, the youngest age group (4–5-month-old) Thy1-APPL1 mice did not show an increase in rab5-GTP early endosome number or size compared with non-Tg, although APPL1 is overexpressed at this age (Fig. 1*D*,*E*). While early endosome size and, correspondingly, early endosome area/cell were increased in all of the older age groups, the number of early endosomes was not found to be increased until the oldest cohort of Thy1-APPL1 mice (Fig. 2*B*, age 22–23 months). Age-dependent endosome alterations were also demonstrated in APOE4 mice (Nuriel et al., 2017) and DS mouse models (Jiang et al., 2016), suggesting that endosome homeostasis may be maintained at younger ages despite a perturbation that ultimately drives endosomal dysfunction as the brain ages.

The endosomal enlargement due to APPL1 overexpression was confirmed by EM in 12–13-month-old Thy1-APPL1 compared with non-Tg mice, where both endosomal size and circumference were significantly increased in layer V cells of prefrontal cortex (Fig. 2*C*,*E*; *n* = 7 for non-Tg and Thy1-APPL1 mice, *t*_(12)_ = 3.64, *p* = 0.0034; *t*_(12)_ = 4.239, *p* = 0.0012 for size and circumference, respectively; two-tailed, unpaired *t* test) and the CA1 region of the hippocampus (Fig. 2*D*,*E*; *n* = 4 for non-Tg and Thy1-APPL1 mice, *t*_(6)_ = 3.327, *p* = 0.0173; *t*_(6)_ = 3.83, *p* = 0.00866 for size and circumference, respectively; two-tailed, unpaired *t* test). Increased association of APPL1 with rab5-positive endosomes has been previously shown in cortical neurons from both AD and DS human brain (Kim et al., 2016), consistent with the increased colocalization of APPL1 and rab5-GTP seen in the Thy1-APPL1 mice (Fig. 2*A*). To further substantiate this colocalization at the early endosome, we performed immuno-EM of cortical and CA1 hippocampal brain sections of 12–13-month-old mice using a rabbit anti-rab5 and a murine anti-APPL1 antibody, followed by species-specific gold-conjugated secondary antibodies (anti-rabbit, 6 nm, green colored; anti-mouse, 10 nm, red colored). EM images from fields containing dendritic profiles were collected and the number of gold particle-positive endosomal vesicles was manually counted. The ratio of rab5/APPL1 double-positive endosomes versus total rab5-positive endosomes was significantly higher in Thy1-APPL1 compared with non-Tg mice in both brain regions (Fig. 2*F*,*G*; *n* = 3 for non-Tg and Thy1-APPL1 mice, *t*_(4)_ = 3.492, *p* = 0.0251; *t*_(4)_ = 2.825, *p* = 0.0476 for cortex and CA1, respectively; two-tailed, unpaired *t* test).

### Endocytic abnormalities in hippocampal synaptosomes of Thy1-APPL1 mice

**Figure 3.**
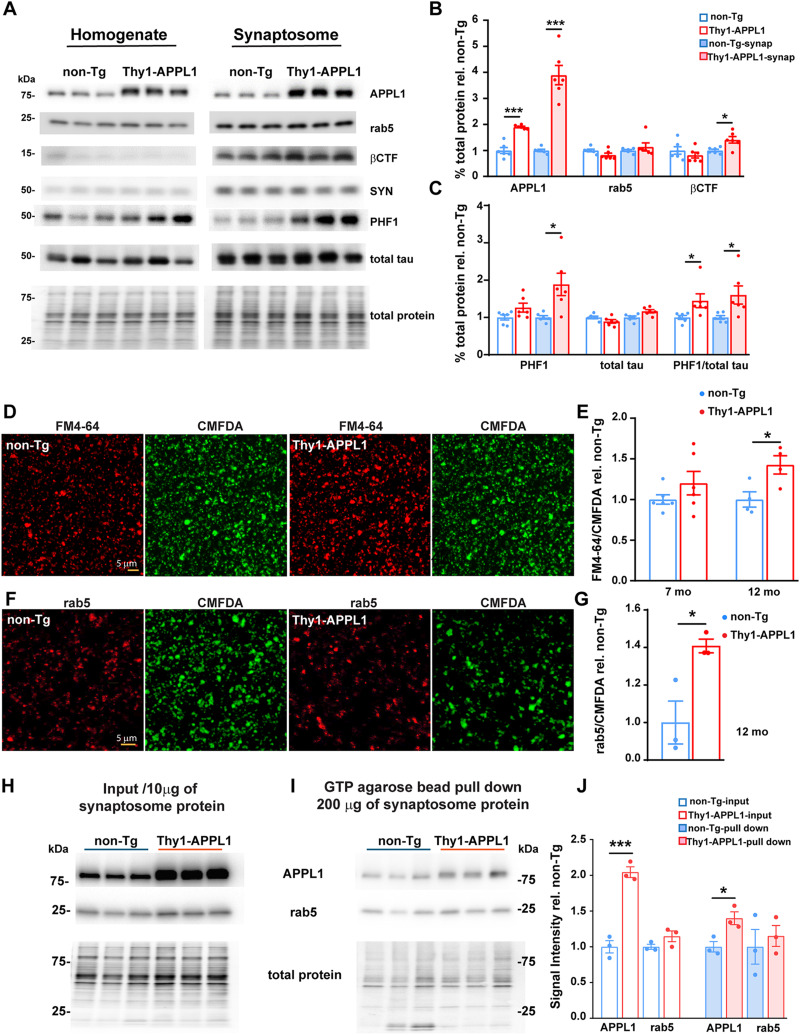
Western blot analysis and synaptic vesicle endocytosis (SVE) revealed the abnormalities in hippocampal synaptosomes of Thy1-APPL1 mice. ***A***, Representative Western blots from one of the two experiments showing various protein markers in hippocampal homogenates and hippocampal synaptosome preparations from non-Tg and Thy1-APPL1 mice (*n* = 6 each genotype). Quantitation of these Western blots (***B***, ***C***) shows significantly higher levels of APPL1, βCTF, PHF1, and the ratio of PHF1/total tau in synaptosomes of Thy1-APPL1 compared with non-Tg mice at 12–13 months of age. ***D***, Representative fluorescent images of internalized FM4-64 (red) with corresponding CMFDA labeling of total synaptosomes (green) using hippocampal synaptosomes prepared from non-Tg and Thy1-APPL1 mice at 12–13 months of age. ***E***, The ratio of internalized FM4-64 to CMDFA was significantly increased in hippocampal synaptosomes of Thy1-APPL1 mice at 12–13 months, but not in 7 months of age, indicating elevated endocytosis in older Thy1-APPL1 mice compared with non-Tg. ***F***, Representative images of CMFDA (green) labeling followed by anti-rab5 (red) immunolabeling and (***G***) quantification of the intensity of rab5 to CMFDA showing an increase in rab5 immunosignal per CMFDA-labeled synaptosome in hippocampal synaptosomes of Thy1-APPL1 mice at 12–13 months of age. Scale bar, 5 µm, *p < 0.05, ***p < 0.001, two-tailed, unpaired *t* test. Data shown as mean ± SEM. Western blots of hippocampal synaptosome (10 µg of synaptosome protein, input; ***H***), and GTP-agarose pull-down of hippocampal synaptosome (200 µg of synaptosome protein) from non-Tg and Thy1-APPL1 mice (***I***) probed with anti-APPL1 and anti-rab5 antibodies, total protein stained by Revert 700 Total Protein Stain are also shown at the bottom of ***H*** and ***I***. The APPL1 and rab5 band density in both input and pull-down against total protein in relation with non-Tg are presented in ***J***, *p < 0.05, ***p < 0.001, one-way ANOVA. Data is shown as mean ± SEM.

Given that our previous studies demonstrated that rab5 hyperactivation leads to synaptic dysfunction and deficits in memory and learning (Kaur et al., 2014; Jiang et al., 2016; Pensalfini et al., 2020) and that endocytic activity plays a critical role in synaptic function (Overhoff et al., 2021), we sought to confirm that the overexpression of APPL1 in the Thy1-APPL1 mouse leads to differences in endocytic function in isolated synaptosomes. Independent of genotype, synapse enrichment was confirmed by the higher level of synaptophysin in the synaptosome preparations compared with hippocampal homogenates (Fig. 3*A* and additional data not shown; *n* = 6 per genotype, at the age of 12–13 months). Consistent with the neuronal expression of APPL1 in the transgenic mice (Fig. 1*C*), the increase of APPL1 protein in the synaptosomes prepared from Thy1-APPL1 mice was found to be greater than that seen in overall brain homogenate (Fig. 3*A*,*B*, 3.9× of non-Tg in synaptosomes compared with 1.9× in homogenate, *n* = 6, *t*_(10)_ = 5.36, *p* = 0.000319, two-tailed, unpaired *t* test). Total rab5 protein levels in both homogenates (also in Fig. 1*D*,*E*) and synaptosomes were unaffected by APPL1 overexpression (Fig. 3*A*,*B*). While APP-βCTF levels were similar in Thy1-APPL1 and non-Tg mouse brain homogenate, APPL1 overexpression led to an increase in APP-βCTF levels within synaptosomes (Fig. 3*A*,*B*; *n* = 6 per genotype; *t*_(10)_ = 3.054, *p* = 0.0122; two-tailed, unpaired *t* test). Given that the APP-βCTF is the immediate precursor of Aβ, we determined brain Aβ levels using a sandwich ELISA that detects the endogenous, murine Aβ (Morales-Corraliza et al., 2009; Jiang et al., 2016) in 7–9 and 12–13-month-old mice. The levels of brain Aβ40 and Aβ42 were found to be unchanged when comparing Thy1-APPL1 with non-Tg mice brain homogenate (data not shown).

Phosphorylated tau (identified with PHF1; Pensalfini et al., 2020) was also increased in hippocampal synaptosomes of Thy1-APPL1 mice compared with non-Tg controls (Fig. 3*A*,*C*; *n* = 6 for per genotype; *t*_(10)_ = 2.922, *p* = 0.0152 for synaptosome PHF1; *t*_(10)_ = 2.309, *p* = 0.0436 for PHF1/total tau in brain homogenate; *t*_(10)_ = 2.432, *p* = 0.0353 for PHF1/total tau in synaptosome homogenate; two-tailed, unpaired *t* test), an increase in PHF1 reactivity comparable with the increase seen following neuronal rab5 overexpression (Pensalfini et al., 2020). These findings in Thy1-APPL1 mouse further argue that endosomal pathway disruption can contribute to tau pathobiology in AD (Knopman et al., 2021).

To directly investigate whether endocytosis was altered by the increase in APPL1 expression in the Thy1-APPL1 mice, total synaptosomes were identified with the fluorescent dye CMFDA (Daniel et al., 2012; Sellgren et al., 2019). Ex vivo synaptosome endocytic uptake was determined using a second fluorescent marker, FM4-64, following its internalization (Daniel et al., 2012; Sellgren et al., 2019). Confocal images of both FM4-64 and CMFDA were collected and the ratio of FM4-64 to CMFDA in synaptosome was quantified. A significantly greater ratio of FM4-64/CMFDA was observed in hippocampal synaptosomes isolated from Thy1-APPL1 compared with non-Tg mice at 12 months of age (Fig. 3*D*,*E*, *n* = 4 for non-Tg and Thy1-APPL1 mice; *t*_(6)_ = 2.886, *p* = 0.0278; two-tailed, unpaired *t* test), while the ratios of FM4-64/CMFDA remain unchanged between non-Tg and Thy1-APPL1 mice at the age of 7 months (Fig. 3*E*, *n* = 6; *t*_(10)_ = 1.294, *p* = 0.225; two-tailed, unpaired *t* test). This age-dependent increase in synaptosomal endocytic uptake is consistent with the age-dependent increase in neuronal early endosome size seen in the Thy1-APPL1 mice (Fig. 2*B*). As determined by immunocytochemistry using an anti-rab5 antibody (Abcam, 1;200), a significantly higher ratio of rab5/CMDFA was also found in Thy1-APPL1 mice at the age of 12 months (Fig. 5*F*,*G*, *n* = 3 for non-Tg and Thy1-hAPPL1 mice; *t*_(4)_ = 3.403, *p* = 0.0272; two-tailed, unpaired *t* test), supporting the idea that rab5 endosomal recruitment in the Thy1-APPL1 mouse leads to greater endocytosis. These findings support our hypothesis that APPL1 acts as a molecular interface connecting rab5 and APP-βCTFs at the early endosome.

We and others have previously used GTP-agarose affinity columns to isolate rab5 from brain tissue and cells (Xu et al., 2016; Fang et al., 2017; Pensalfini et al., 2020). In this study, we used GTP-agarose affinity to isolate rab proteins and to coisolate rab5-associated APPL1 (Miaczynska et al., 2004; Mao et al., 2006; Zhu et al., 2007; Hiragi et al., 2022) to confirm within neurons an increased association of APPL1 and rab5 in the Thy1-APPL1 mouse compared with non-Tg. Consistent with the increased level seen in brain homogenate (Fig. 1), APPL1 was more abundant in the synaptosome preparation from Thy1-APPL1 mice compared with non-Tg (Fig. 3*H*,*J*). Following GTP-agarose isolation of rab proteins, APPL1 was coisolated from both Thy1-APPL1 and non-Tg mice. However, more APPL1 in the GTP-agarose pull-down were seen in the Thy1-APPL1 synaptosomes than in those prepared from non-Tg mice (Fig. 3*I*,*J*; *n* = 3 for non-Tg and Thy1-APPL1 mice; for APPL1 input, *t*_(8)_ = 9.081, *p* < 0.0001; for APPL1 pull-down, *t*_(8)_ = 3.472, *p* = 0.0168; one-way ANOVA). Similar levels of rab5 were isolated from both genotypes using the GTP-agarose pull-down, consistent with our findings showing that total rab5 protein levels are not increased in the Thy1-APPL1 mouse, both in brain homogenates and isolated synaptosomes (Fig. 3*A*,*B*,*H*,*J*). The greater amount of APPL1 isolated from Thy1-APPL1 mouse synaptosomes using the rab GTP-affinity column is consistent with previous findings of APPL1 and rab5 directly interacting (Miaczynska et al., 2004; Mao et al., 2006; Zhu et al., 2007; Hiragi et al., 2022) and supports our interpretation that the synaptosomal endocytic differences seen in Thy1-APPL1 result from APPL1 overexpression leading to enhanced rab5 activation due to an enhanced interaction between the two molecules.

### Loss of BFCNs and endosome accumulation within neurons in Thy1-APPL1 mice

**Figure 4.**
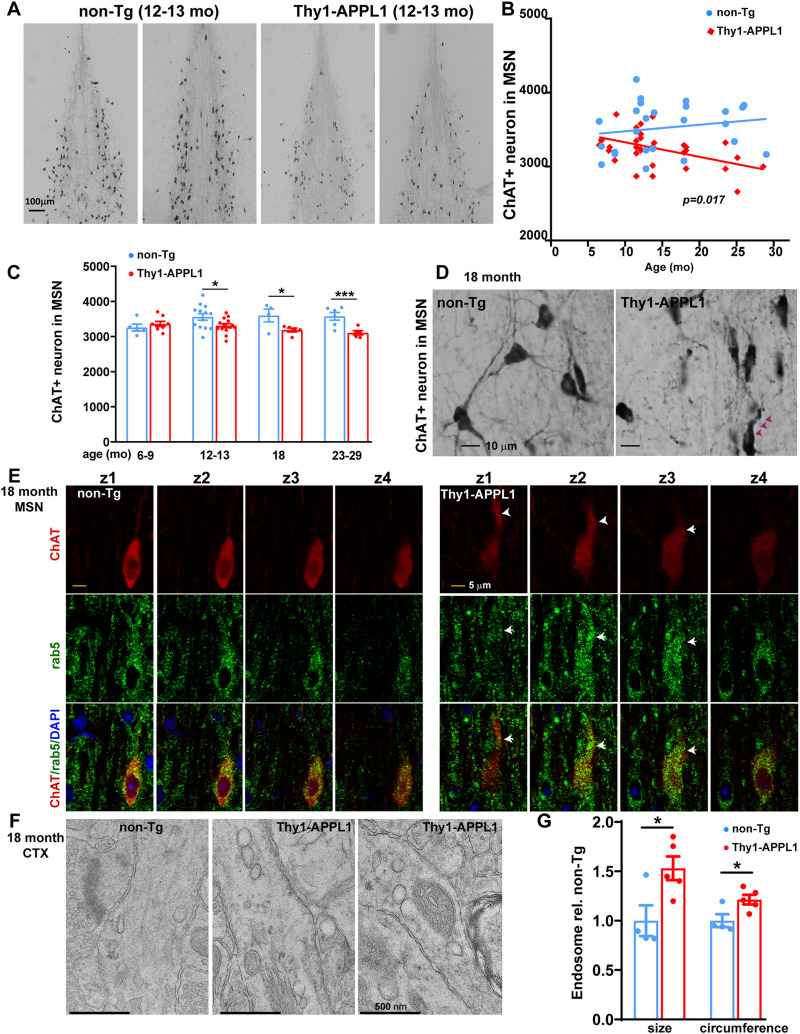
Loss of cholinergic neurons in the medial septal nucleus (MSN) of Thy1-APPL1 mice and accumulation of endosomes in Thy1-APPL1 mice. ***A***, Representative light microscopy images comparing ChAT-immunoreactive neurons in the MSN of non-Tg and Thy1-APPL1 mice at 12–13 months of age. Scale bar, 100 µm. ***B***, Plots of number of ChAT^+^ neurons counted stereologically versus age of mice showing significant difference between the slopes of non-Tg and Thy1-APPL1 mice (*F*_(1,55)_ = 6.01, *p* = 0.017, linear regression). ***C***, Number of ChAT-immunoreactive neurons in the MSN of non-Tg and Thy1-APPL1 mice at the indicated ages (*n* = 42 for non-Tg and 41 for Thy1-APPL1 mice; quantitative stereology as described in Materials and Methods). ***D***, Representative images of ChAT-immunoreactive neurons at higher magnification in 18-month-old non-Tg and Thy1-APPL1 mice (arrowheads indicate dystrophic neurites). Scale bar, 5 µm. ***E***, Representative *z*-stacked series fluorescence images of ChAT- (red) and rab5-immunoreactive (green) neuron in the MSN of a non-Tg and Thy1-APPL1 mice at 18 months of age (arrowheads indicating a dystrophic neurite). Scale bar, 5 µm. *p < 0.05, ***p < 0.001, two-tailed, unpaired *t* test. Data presented as mean ± SEM. ***F***, Representative EM images of 18-month-old mice showing an accumulation of endosomes in cortical dendrites of Thy1-APPL1 mice compared with non-Tg mice (scale bar, 500 nm). ***G***, Quantification of average size and circumference of endosomes in the cortical dendritic area of non-Tg and Thy1-APPL1 mice at the age of 18 months (*n* = 4 for non-Tg and *n* = 5 for Thy1-APPL1 mice; 60 cortical section images/mouse, a total of 982 endosomes were counted in Thy1-APPL1mice, with an average of 196 endosomes/mouse counted. 630 total endosomes were counted from non-Tg mice, with averaging 157 endosomes/mouse), *p < 0.05, two-tailed, unpaired *t* test. Data shown as mean ± SEM.

Neuronal endosomal dysfunction drives the loss of cholinergic neurons in the MSN due to a loss of trophic support mediated by signaling endosomes (Salehi et al., 2003; Howe and Mobley, 2004; Jiang et al., 2016, 2022; Xu et al., 2018; Chen and Mobley, 2019; Pensalfini et al., 2020). Consistent with the endosomal alterations seen in the Thy1-APPL1 model, the number of ChAT-immunoreactive neurons in the MSN of Thy1-APPL1 mice showed an age-dependent loss compared with non-Tg mice (Fig. 4*A*,*B*, *n* = 42 for total of non-Tg mice and *n* = 41 for total of Thy1-APPL1 mice; *F*_(1,55)_ = 6.01, *p* = 0.017, linear regression), with reduced numbers of cholinergic neurons first detected at 12 months of age (Fig. 4*C*, *t*_(25)_ = 2.316, *p* = 0.0291 for 12–13-month-old mice; *t*_(7)_ = 2.399, *p* = 0.0475 for 18-month-old mice; *t*_(9)_ = 3.482, *p* = 0.00692 for mice over 23-month-old; two-tailed, unpaired *t* test).

**Figure 5.**
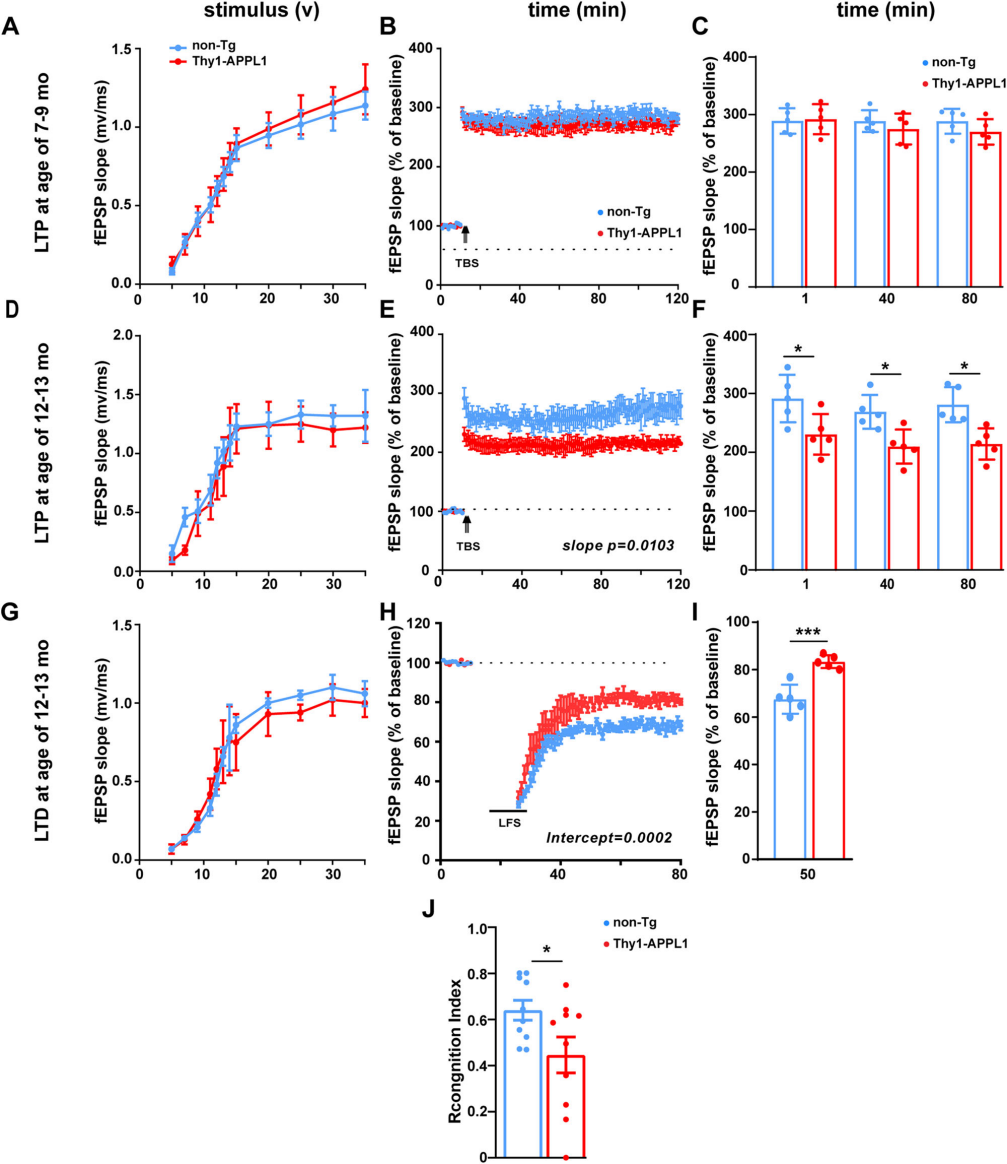
Age-related impairments of synaptic plasticity and hippocampal-dependent memory deficit in Thy1-APPL1 mice. ***A–C***, Non-Tg and Thy1-APPL1 mice at 7–9 months of age (*n* = 5 for both genotype), input/output relationship plots (***A***), LTP by theta-burst stimulation (TBS) in the Schaffer collateral synapses (CA3-CA1) of hippocampal slices (***B***), and averages of fEPSP slopes at 1, 40, and 80 min following tetanic stimulation (***C***) of the hippocampal slices showing no differences between non-Tg and Thy1-APPL1 mice. ***D–F***, Non-Tg and Thy1-APPL1 mice at 12–13 months of age (*n* = 5 for both genotype), input/output relationship plots (***D***), LTP induced by TBS in the Schaffer collateral synapses (CA3-CA1) in hippocampal slices (***E***), and averages of fEPSP slopes at 1, 40, and 80 min (***F***) showing significant reduction in Thy1-APPL1 mice (*F*_(1,236)_ = 6.696, *p* = 0.0103 for the slope, linear regression). ***G–I***, Non-Tg and Thy1-APPL1 mice at 12–13 months of age (*n* = 5 for both genotypes), input/output relationship plots (***G***), LTD induced by low-frequency stimulation (LFS; ***H***) in hippocampal slices of Thy1-APPL1 (*F*_(1,127)_ = 14.83, *p* = 0.0002 for the intercepts, linear regression), and averages of fEPSP slopes at 50 min following LFS induction (***I***) in the hippocampal slices did not show expected reduction in Thy1-APPL1 mice. ***J***, Recognition index at 3 h after familiarization indicated the memory deficit in Thy1-APPL1 mice at 12–13 months of age (*t*_(18)_ = 2.187, *p* = 0.042). *p < 0.05, ***p < 0.001, two-tailed, unpaired *t* test. Data is shown as mean ± SEM.

Abnormal, tortuous proximal dendrites and shrunken cholinergic cell soma (Fig. 4*D*, arrowheads) were also seen in the MSN neurons of aged Thy1-APPL1 compared with non-Tg mice (18 months), as has been reported in multiple AD-like models with endosomal alterations (Cataldo et al., 2000, 2003; Salehi et al., 2006; Choi et al., 2009; Jiang et al., 2016, 2022; Pensalfini et al., 2020). Figure 4*E* shows representative *Z*-stacked images of immunolabeling rab5-positive early endosomes within cholinergic neurons in a Thy1-APPL1 compared with a non-Tg mouse, both at 18 months of age. Accumulation of early endosomes (arrowheads) was apparent in the ChAT-immunoreactive cholinergic neurons of the Thy1-APPL1 relative to non-Tg mice. Representative EM images of 18-month-old mice also demonstrated an accumulation of endosomes (Fig. 4*F*) as well as a significant endosome size increase in cortical dendritic areas of Thy1-APPL1 mice compared with non-Tg (Fig. 4*G*; *n* = 4 for non-Tg, and *n* = 5 for Thy1-APPL1 mice; *t*_(7)_ = 2.753, *p* = 0.0284 for the size; *t*_(7)_ = 2.701, *p* = 0.0306 for the circumference). This EM finding is consistent with the previous immunolabeling documenting an increase in active rab5 early endosomes in neurons of the Thy1-APPL1 mice (Fig. 2).

### Hippocampal synaptic plasticity impairments in Thy1-APPL1 mice

Alteration of hippocampal synaptic plasticity has been shown in AD-related mouse models (Marchetti and Marie, 2011; Dietrich et al., 2018), and APPL1 has been shown to be a regulator of hippocampal synaptic plasticity (Fernandez-Monreal et al., 2016; Wang et al., 2016; Formolo et al., 2022; Hua et al., 2023). We have also previously demonstrated that overactivation of rab5 resulting from neuronal rab5 overexpression leads to hippocampal-dependent memory deficits (Pensalfini et al., 2020). Long-lasting changes in synaptic plasticity measured as LTP and LTD are key mechanisms upon which learning and long-term memory are built (Avchalumov and Mandyam, 2021). Greater rab5 activation has been linked with both LTP deficit (Pensalfini et al., 2020) and LTD impairments (Hausser and Schlett, 2019; Pensalfini et al., 2020). To determine whether APPL1 overexpression in the Thy1-APPL1 mouse altered hippocampal plasticity, we measured LTP and LTD of CA1hippocampal slices of Thy1-APPL1 and non-Tg mice (Fig. 5; *n* = 5 for both genotypes at ages 7–9 and 12–13 months). In the younger age cohort (7–9 months), Thy1-APPL1 mice did not show differences compared with non-Tg in LTP following TBS stimulation (Fig. 5*A–C*). However, we found a significant LTP impairment in 12–13-month-old Thy1-APPL1 mice [Fig. 5*E* (*F*_(1,236)_ = 6.696, *p* = 0.0103 for the slope, linear regression) and Fig. 5*F* (*t*_(8)_ = 2.565, *p* = 0.0334 for 1 min time point; *t*_(8)_ = 3.237, *p* = 0.0237 for 40 min time point; *t*_(8)_ = 3.722, *p* = 0.0175 for 80 min time point; two-tailed, unpaired multiple *t* test)], without apparent difference in their basal neurotransmission between the genotypes (Fig. 5*D*). Furthermore, postsynaptic potential (fEPSP) slopes indicated suppressed LTD upon induction by low frequency stimulation (1 Hz for 900 s) in 12–13-month-old Thy1-APPL1 mice compared with their age-matched non-Tg littermates [Fig. 5*H* (*F*_(1,127)_ = 14.83, *p* = 0.0002 for the intercepts, linear regression) and Fig. 5*I* (*t*_(8)_ = 5.277, *p* = 0.000749 for 50 min time point; two-tailed, unpaired *t* test)]. Thus, we show both loss of BFCNs and disruption of hippocampal CA1 LTP and LTD as the Thy1-APPL1 mice age. Consistent with these electrophysiological findings, hippocampal-dependent memory assessed by NOR showed impairment in Thy1-APPL1 mice at 12–13 months of age (Fig. 5*J*; *n* = 10 for non-Tg and Thy1-APPL1 mice; *t*_(18)_ = 2.187, *p* = 0.042; two-tailed, unpaired *t* test).

## Discussion

Our findings provide strong in vivo evidence implicating APPL1 as a key molecular intermediate in a pathogenic cascade propelled by causative and risk genes for AD that lead to abnormal rab5 early endosome signaling and neurodegeneration of BFCN, preceding and predicting the cortical spread of Alzheimer's pathology (Schmitz and Nathan Spreng, 2016; Schmitz et al., 2018). This cascade is further linked to the decline of memory (Alam and Nixon, 2023), and its detection in prodromal AD (Rozalem Aranha et al., 2023) coincides with the early emergence of signature early endosome enlargement, reflecting rab5 hyperactivation. Clinical trials aimed at pharmacologically attenuating rab5 overactivation have shown encouraging improvement in measures of cholinergic function in Lewy body dementia, a disorder in which BFCN loss of function contributes prominently to the clinical presentation (Jiang et al., 2022). The fidelity with which our Thy1-APPL1 replicates the endosomal dysfunction and neurodegenerative consequences seen in AD (Cataldo et al., 1996, 2000; Nixon, 2017), APP-based mouse models of AD and DS (Salehi et al., 2006; Jiang et al., 2010, 2016), and a mouse model of rab5 overactivation (Pensalfini et al., 2020), underscores the important contribution of APPL1 to mediating pathogenic actions of rab5, as suggested by earlier molecular studies of APPL1 in primary neuron cultures (Kim et al., 2016; Xu et al., 2016).

**Figure 6.**
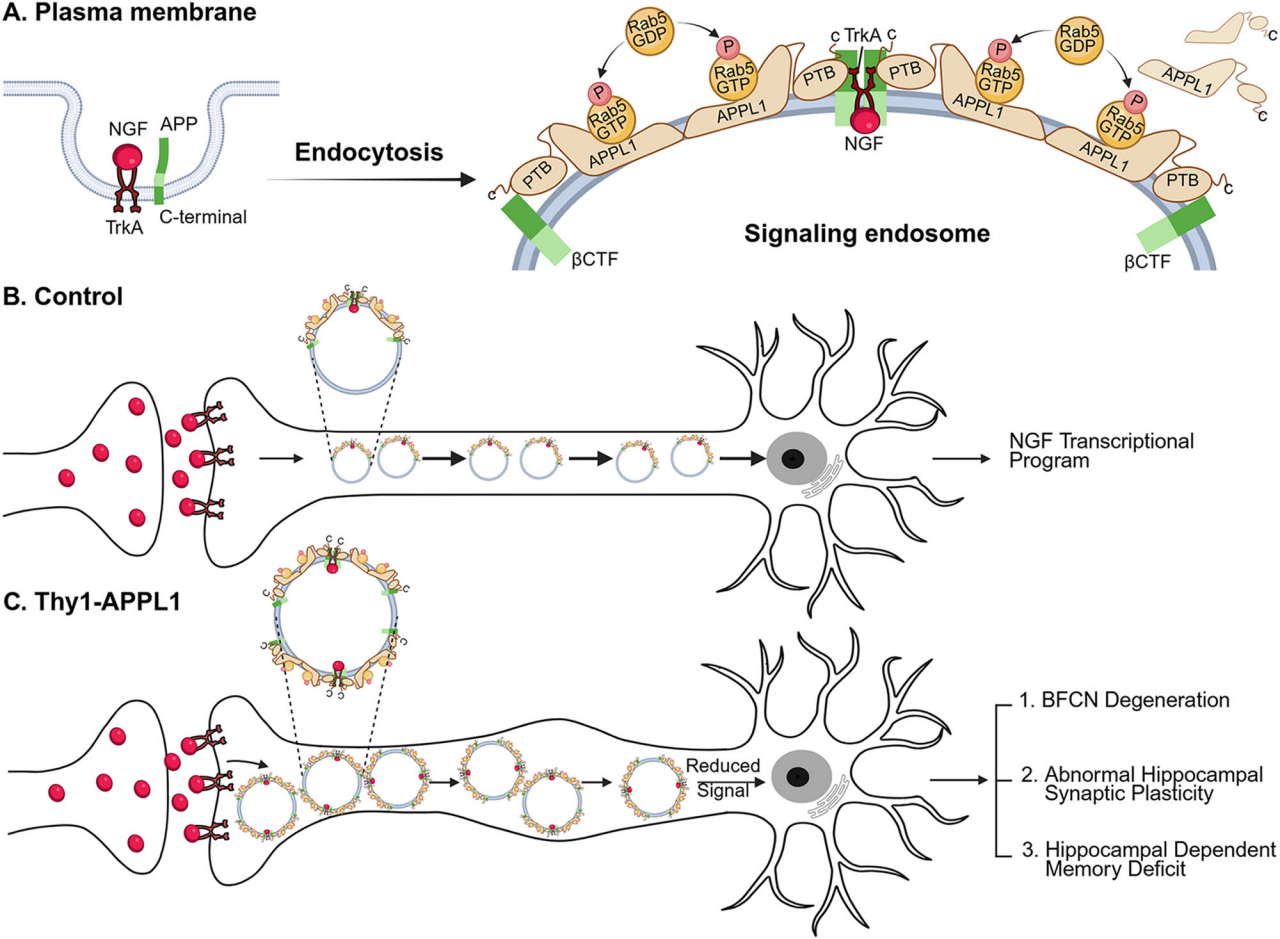
NGF retrograde signaling and involvement of APPL1 in healthy neurons and after a rise in neuronal APPL1 levels related to Alzheimer's disease in APPL1 overexpressing neurons. Schematic diagram depicts (***A***) the endocytosis of APP, NGF, and its receptor TrkA into a rab5 early endosome. TrkB mediation of BDNF signaling by APPL1 (data not shown) is considered to follow a similar sequence. ***B***, Normal NGF signaling is facilitated by recruitment of APPL1, a direct TrkA ligand and adaptor for other signaling molecules mediating retrograde transport of a maturing endosome carrying the NGF signal to the nucleus to activate a neurotrophic transcriptional program supporting functioning of ChAT neurons and other NGF targets. In AD (not shown; see main text), abnormally elevated APP-βCTF levels arising via multiple possible mechanisms raise levels of the activated form of rab5 (rab5-GTP) on endosomal membranes, in part by recruiting more APPL1 to the endosome via the phosphotyrosine binding (PTB) domain of APPL1 (Kim et al., 2016). APPL1's greater affinity for rab5-GTP prolongs association of this activated form on the endosome, thus promoting a pathogenic rab5 hyperactivation leading to increased endocytosis, early endosomal fusion, and endosome enlargement, as depicted in ***C***. ***C***, Moderately elevating APPL1 selectively in neurons of Thy1-APPL1 mice phenocopies mouse models of APP-βCTF elevation or rab5 overexpression with respect to rab5 hyperactivation, abnormal endosome enlargement, stasis of endosome transport, synaptic plasticity deficits, and basal forebrain cholinergic neurodegeneration. These pathological effects, as observed in AD brain, also reflect impaired NGF/TrkA signaling and decreased expression of genes for neuronal survival, growth and differentiation (Niewiadomska et al., 2011; Xu et al., 2018). Further information is provided in the text and in more detail in reviews (Nixon, 2017; Nixon and Rubinsztein, 2024).

Endogenous APP-βCTF at modestly elevated levels in DS mouse models enlarges early endosomes, impairing neurotrophic function via retrogradely transported signaling endosomes (Salehi et al., 2006; Xu et al., 2016) and early degeneration of BFCNs (Jiang et al., 2016, 2022; Fig. 6). Evidence in cell models shows overexpressed APPL1 binds to APP-βCTFs, enlarged early endosomes, and slows axonal transport of endosomes in proportion to the increase in endosome size (Kim et al., 2016). Conversely, in DS fibroblasts, which have enlarged early endosomes due to increased APP-βCTF levels, treating with siRNA to reduce APPL1 levels restores early endosome morphology and function to that seen in diploid cells (Kim et al., 2016). Both APP-βCTF and APPL1 levels are increased in human AD brain (Kim et al., 2016; Johnson et al., 2022), as are the colocalization of APPL1 with rab5-positive endosomes (Kim et al., 2016). Our Thy1-APPL1 mouse directly models an increase in APPL1 expression as reported in human AD, showing that such an increase in APPL1 is sufficient to lead to early endosomal alterations and functional changes in neuron.

Given the foregoing in vitro evidence in APP overexpression systems and DS fibroblasts (Jiang et al., 2010; Kim et al., 2016), we undertook the current study to further dissect APPL1's roles in vivo. APPL1 recruitment to rab5 endosomes was shown in cell models to be necessary for APP-βCTF-mediated rab5 overactivation, an outcome consistent with APPL1's action as a stabilizer of the active rab5-GTP complex on endosomes (Kim et al., 2016). With its expression in brain approximately doubled in Thy1-APPL1 mice, APPL1 recapitulated an aging-dependent onset of rab5 overactivation, early endosome swelling (Cataldo et al., 1997; Jiang et al., 2016; Kim et al., 2016; Mathews and Levy, 2019; Pensalfini et al., 2020), and loss of BFCNs, a phenotype invariably seen in AD brain and the various AD models that replicate AD-related early endosome dysfunction (Cataldo et al., 1997; Jiang et al., 2016; Kim et al., 2016; Pensalfini et al., 2020). In the Thy1-APPL1 model, early endosome alterations and these downstream effects were aging dependent, with early endosomes beginning to enlarge at 7–9 months of age and prior to BFCN loss at 12–13 months of age. Mouse disease models typically show measurable BFCN degeneration months following detectable endosomal alterations (Cataldo et al., 2000, 2003; Salehi et al., 2006; Choi et al., 2009; Jiang et al., 2016, 2022; Pensalfini et al., 2020). Changes in LTP and LTD were also aging dependent and initially detected at 12–13 months of age, later than the age when early endosome enlargement in cortical neurons was first seen, consistent that the full impact of dysfunctional endosomes plays out over time. That neuronal early endosome enlargement in the Thy1-APPL1 mouse is not seen at 4–5 months of age but detected later is consistent with other models in which the neuronal early endosome appears to be resilient in the younger brain, while this homeostatic ability is lost during aging (Nuriel et al., 2017; Mathews and Levy, 2019). Interestingly, in a model with direct rab5 overactivation (Pensalfini et al., 2020), loss of endosomal homeostasis is apparent early, suggesting that disturbances of rab5 regulatory mechanisms, albeit capable of driving endosomal dysfunction through rab5 overactivation, may be tolerated until the brain begins to age.

NGF signaling is essential for the survival of BFCNs (Sofroniew et al., 1990; Holtzman et al., 1992), and loss of NGF signaling leading to cognitive decline is an early feature in AD and DS (Grothe et al., 2012; Schmitz and Nathan Spreng, 2016). NGF is transduced via endocytosis and the retrograde transport and signaling by a APPL1/rab5 “signaling endosome” containing the NGF receptor TrkA (Xu et al., 2016; Villarroel-Campos et al., 2018). Impaired endosome transport and signaling degrade the NGF trophic stimulus to cholinergic neurons (Salehi et al., 2006; Kim et al., 2016; Kulkarni et al., 2022; Fig. 6). Increasing NGF levels can rescue these neurons (Granholm et al., 2000; Eu et al., 2021), while reducing rab5 overactivation (Xu et al., 2016; Jiang et al., 2022) or APP-βCTF levels (Jiang et al., 2016) prevents endosome dysfunction and preserves cholinergic function. Our study therefore offers the possibility of targeting rab5 via modulation of APPL1 to reactivate functional cholinergic neurons and reverse disease progression.

Increased phosphorylated tau, as detected by PHF1, was also measured in synaptosomes isolated from the Thy1-APPL1 mice (Fig. 3). Endosomal pathway alterations are known to impact tau phosphorylation as well as the spread and seeding of pathogenic tau within the brain (Knopman et al., 2021). APPL1 influence on multiple protein kinases involved in axonal transport (e.g., Akt, GSK3ß, MAP kinase; Mitsuuchi et al., 1999; Diggins and Webb, 2017) is also likely to affect tau phosphorylation directly in the Thy1-APPL1 model.

Rab5 is associated with both pre- and postsynaptic vesicles, where rab5 is critical in mediating function by regulating SVE and thus membrane and protein cargo retrieval (Star et al., 2005; Chanaday et al., 2019). These are also endosomal functions vulnerable in neurodegenerative diseases (Bonnycastle et al., 2021; Harper and Smillie, 2021; Bellucci et al., 2022). Synaptosomes containing numerous synaptic vesicles have been used as an ex vivo system to study synaptic dysfunctions in AD and other neurodegenerative diseases (Evans, 2015; Jhou and Tai, 2017; Ahmad and Liu, 2020; Griffiths and Grant, 2023). We observed that increased APPL1 expression induces synaptosome endocytosis, consistent with its activating rab5 on neuronal endosomes. Both knockdown mediated by siRNA and an APPL1 functional knockdown mediated by inhibitory peptides in vitro show that APPL1 is necessary for normal hippocampal LTP and LTD (Fernandez-Monreal et al., 2016; Wang et al., 2016; Wu et al., 2020; Formolo et al., 2022; Hua et al., 2023). We find that APPL1 overexpression in vivo also impacts hippocampal electrophysiology. Given APPL1's functions at the early endosome, both under- and overexpression of APPL1 are likely to perturb early endosome signaling as well as the formation and cargo acquisition of early endosomes through endocytosis (Diggins and Webb, 2017). Overexpression of APPL1 in the Thy1-APPL1 mouse increased rab5 activity, driving synaptic endocytosis. It is also likely to lead to entrapment of endocytic cargo, which enlarges early endosomes preventing appropriate synaptic vesicle recycling and trafficking. The increase of APP-βCTFs seen in the synaptosomes of Thy1-APPL1 mouse is also consistent with endocytosed cargo being entrapped in early endosomes. BACE1 cleavage of APP to generate APP-βCTFs occurs in endocytic compartments, and increasing the uptake and dwell time of APP within endosomes increases APP-βCTF generation (De Strooper and Karran, 2016). Abundant APPL1 at endosomes, given its interaction with endosomal APP-βCTFs, is likely to increase the dwell time of the rab5/APPL1/APP-βCTF complex, further accentuating rab5 activation.

Among its various neuronal signaling functions (Lin et al., 2006; Deepa and Dong, 2009; Lee et al., 2011; Hupalowska et al., 2012), APPL1 acts as a molecular link between APP-βCTF and rab5 (Kim et al., 2016), a critical crossroads for pathways that converge on the early endosome during AD pathobiology. The Thy1-APPL1 mouse highlights the important role APPL1 can play in transducing APP-βCTF-signaling in AD to rab5, thus regulating early endosome function, growth factor signaling, and cholinergic neuronal functioning. We now show that overexpression of APPL1 leads to phenotypes similar to those that result from the direct overactivation of rab5 (Pensalfini et al., 2020). Whether other risk factors for AD-driven early endosome and synaptic dysfunction, such as APOE4, BIN1, CD2AP, PICALM, RIN3, SORL1, and others (Nixon, 2017; Perdigão et al., 2020), act through APPL1 is unknown; nevertheless, the Thy1-APPL1 model further demonstrates that multiple molecular mechanisms leading to AD-like early endosome dysfunction are known to converge on rab5 and its regulation.
